# Neoadjuvant Atezolizumab and Chemotherapy for Non‐Squamous Non‐Small Cell Lung Cancer: Efficacy and Safety Results of an Open‐Label, Single‐Arm, Phase II Trial

**DOI:** 10.1002/ijc.70508

**Published:** 2026-04-19

**Authors:** Benedikt Niedermaier, Tilmann Bochtler, Florian Eichhorn, Raffaella Griffo, Laura V. Klotz, Michael Allgäuer, Albrecht Stenzinger, Helge Bischoff, Marc A. Schneider, Petros Christopoulos, Uwe Haberkorn, Claus Peter Heussel, Inka Zoernig, Felix J. F. Herth, Michael Thomas, Alexander Rölle, Hauke Winter, Axel Benner, Dirk Jaeger, Martin E. Eichhorn

**Affiliations:** ^1^ Department of Thoracic Surgery, Thoraxklinik Heidelberg University Hospital Heidelberg Germany; ^2^ Translational Lung Research Center (TLRC), Member of the German Center for Lung Research (DZL) Heidelberg Germany; ^3^ Division of Systems Biology of Signal Transduction German Cancer Research Center (DKFZ), DKFZ‐ZMBH Alliance Heidelberg Germany; ^4^ Department of Medical Oncology Heidelberg University Hospital, Medical Faculty Heidelberg, Heidelberg University Heidelberg Germany; ^5^ Department of Thoracic Oncology, Thoraxklinik Heidelberg University Hospital Heidelberg Germany; ^6^ Institute of Pathology, Heidelberg University Hospital Heidelberg Germany; ^7^ Translational Research Unit (STF), Thoraxklinik Heidelberg University Hospital Heidelberg Germany; ^8^ Clinical Cooperation Unit Nuclear Medicine, German Cancer Research Center (DKFZ) Heidelberg Germany; ^9^ Department of Nuclear Medicine Heidelberg University Hospital Heidelberg Germany; ^10^ Department of Diagnostic and Interventional Radiology With Nuclear Medicine, Thoraxklinik Heidelberg University Hospital Heidelberg Germany; ^11^ Department of Diagnostic and Interventional Radiology Heidelberg University Hospital Heidelberg Germany; ^12^ Clinical Cooperation Unit Applied Tumor Immunity, National Center for Tumor Diseases, German Cancer Research Center Heidelberg Germany; ^13^ Center for Quantitative Analysis of Molecular and Cellular Biosystems (Bioquant) Heidelberg University Heidelberg Germany; ^14^ National Center for Tumor Diseases (NCT) Heidelberg Heidelberg Germany; ^15^ Department of Pulmonology and Critical Care Medicine, Thoraxklinik Heidelberg University Hospital Heidelberg Germany; ^16^ Division of Biostatistics German Cancer Research Center (DKFZ) Heidelberg Germany

**Keywords:** immunotherapy, lung cancer, neoadjuvant, pathologic response

## Abstract

Immune checkpoint inhibitors have shown promising results in the neoadjuvant treatment of resectable non‐small cell lung cancer. This open‐label, single‐arm, prospective, monocentric trial evaluated the efficacy and safety of neoadjuvant atezolizumab plus carboplatin/nab‐paclitaxel in patients with resectable non‐squamous non‐small cell lung cancer. Patients with previously untreated, pathologically confirmed, non‐squamous non‐small cell lung cancer in stage II, IIIA, and select IIIB (T3N2 only) were treated with atezolizumab and carboplatin/nab‐paclitaxel for 3 cycles followed by curative intent surgery. Major pathologic response (MPR) was defined as primary endpoint. 20 patients with histologically confirmed pulmonary adenocarcinoma in TNM‐stage IIA (*n* = 1, 5%), stage IIB (*n* = 7, 35%), and stage IIIA (*n* = 12, 60%) were enrolled and treated according to the study protocol. 151 treatment‐related adverse events were recorded, and 13 patients (65%) had treatment‐related adverse events of grade 3 or higher. There were no grade 5 events. All patients underwent complete anatomical resection (R0). MPR was observed in 9 patients (45%), including 5 (25%) patients with complete pathological response. The proportion of remaining viable tumor showed a significant but weak association to the relative tumor size change in CT (*p* = 0.018) and the relative change in SUV_max_ (*p* = 0.006). In conclusion, neoadjuvant chemoimmunotherapy with atezolizumab achieved a promising MPR‐rate of 45% while being well tolerated and allowing a safe and complete surgical resection. These results strongly support the further investigation of atezolizumab as preoperative therapy in resectable non‐small cell lung cancer and underline the continued need to develop biomarkers of response.

## Graphical abstract

Surgery plus chemotherapy is a well‐established treatment protocol for resectable, locally advanced non‐small cell lung cancer (NSCLC). Nonetheless, survival gains remain limited, emphasizing the need for more effective treatments. Here, the authors evaluated a novel approach using a neoadjuvant immunotherapy‐chemotherapy regimen consisting of atezolizumab with carboplatin/nab‐paclitaxel, followed by surgery. Major pathologic response (MPR) occurred in 45 percent of patients with resectable non‐squamous NSCLC. One‐quarter of these patients experienced complete responses, and all achieved complete tumor resection despite adverse events. The findings indicate that chemoimmunotherapy is a promising, tolerable preoperative strategy for resectable non‐squamous NSCLC, warranting further research, particularly into predictive biomarkers.

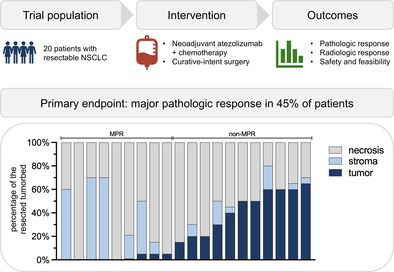

## Abstract (de)

What's new?

Surgery plus chemotherapy is a well‐established treatment protocol for resectable, locally advanced non‐small cell lung cancer (NSCLC). Nonetheless, survival gains remain limited, emphasizing the need for more effective treatments. Here, the authors evaluated a novel approach using a neoadjuvant immunotherapy‐chemotherapy regimen consisting of atezolizumab with carboplatin/nab‐paclitaxel, followed by surgery. Major pathologic response occurred in 45 percent of patients with resectable non‐squamous NSCLC. One‐quarter of these patients experienced complete responses, and all achieved complete tumor resection despite adverse events. The findings indicate that chemoimmunotherapy is a promising, tolerable preoperative strategy for resectable non‐squamous NSCLC, warranting further research, particularly into predictive biomarkers.

**Abbreviations**
AE: Adverse Event
chemoIO: Chemotherapy plus Immunotherapy
CPR: Complete Pathologic Response
CR: Complete Response
CT: Computed Tomography
CTCAE: Common Terminology Criteria for Adverse Events
ECOG‐PS: Eastern Cooperative Oncology Group Performance Status
EORTC: European Organisation for Research and Treatment of Cancer
FDG: Fluorodeoxyglucose
IO: Immunotherapy
IQR: Interquartile Range
irAE: Immune‐Related Adverse Event
MIS: Minimally Invasive Surgery
MPR: Major Pathologic Response
nab‐Paclitaxel: Nanoparticle Albumin‐Bound Paclitaxel
NGS: Next‐Generation Sequencing
NSCLC: Non‐Small Cell Lung Cancer
PBMC: Peripheral Blood Mononuclear Cells
PD: Progressive Disease
PD‐1: Programmed Death‐1
PD‐L1: Programmed Death‐Ligand 1
PET: Positron Emission Tomography
PR: Partial Response
QLQ‐C30: Quality of Life Questionnaire‐Core 30
QLQ‐LC13: Quality of Life Questionnaire‐Lung Cancer 13
R0: Complete Surgical Resection With Negative Margins
RATS: Robotic‐Assisted Thoracic Surgery
RECIST: Response Evaluation Criteria in Solid Tumors
SD: Stable Disease
SUV_max_
: Maximum Standardized Uptake Value
TRAE: Treatment‐Related Adverse Event
UICC: Union for International Cancer Control
VATS: Video‐Assisted Thoracic Surgery

## 1 Introduction

Lung cancer is the leading cause of cancer‐related deaths worldwide, with non‐squamous non‐small cell lung cancer (NSCLC) accounting for 40% to 60% of all lung cancer cases [1, 2]. Surgery is considered the treatment of choice for patients with resectable disease. However, despite complete tumor resection, 30% to 70% of patients will ultimately develop recurrence and die as a result of disease progression, predominantly driven by micrometastatic disease and metastatic recurrence mostly within the first 2 years after surgery [3]. For patients with resectable, locally advanced NSCLC, the combination of surgery with adjuvant or neoadjuvant platinum‐based chemotherapy has only improved survival outcomes by about 5% in comparison to surgery alone [4]. Against this background, the introduction of immunotherapy (IO) has yielded encouraging results, culminating in the recent approval of neoadjuvant and perioperative chemoimmunotherapy targeting the PD1/PD‐L1 pathway as standard therapies for stage IB–IIIA NSCLC in both the United States and Europe [5, 6, 7, 8]. Although neoadjuvant IO alone has been shown to achieve a major pathologic response (MPR) in 20% to 30% of patients, the combination with chemotherapy, referred to as “chemoIO,” has achieved MPR rates of up to 83% in early phase II trials and still above 30% in larger phase III trials [5, 6, 7, 8, 9, 10, 11, 12, 13, 14, 15, 16, 17, 18].

Atezolizumab, a humanized IgG1 monoclonal antibody that targets PD‐L1 and inhibits the interaction between PD‐L1 and its receptors PD‐1 and CD80, has been investigated subordinately in this setting. First, efficacy of chemoIO with atezolizumab was demonstrated in metastatic NSCLC [19]. Building on this, exploratory studies have evaluated the use of atezolizumab in the neoadjuvant setting for resectable NSCLC, reporting encouraging results including MPR rates of 20% as a single agent and 57% in combination with platinum‐based chemotherapy, along with tolerable toxicity and safety [10, 20]. Subsequently, the randomized phase‐III trial IMpower030 was initiated to investigate the effectiveness of neoadjuvant chemoIO with atezolizumab in resectable NSCLC and is currently ongoing [21].

Given this early evidence on the clinical efficacy of atezolizumab, more detailed studies are needed to further confirm the safety and efficacy of this treatment regimen. In this context, the present trial was initiated to explore the efficacy of neoadjuvant atezolizumab plus carboplatin/nab‐paclitaxel for patients with non‐squamous NSCLC as determined by the rate of MPR. Secondary objectives include non‐invasive assessments of tumor response and survival endpoints. Furthermore, the trial design aims to leverage tissue samples from before and after neoadjuvant treatment for exploratory analyses of biomarkers associated with response or resistance to neoadjuvant checkpoint inhibition.

## 2 Methods

### 2.1 Trial Design, Patients and Treatment

**FIGURE 1:**
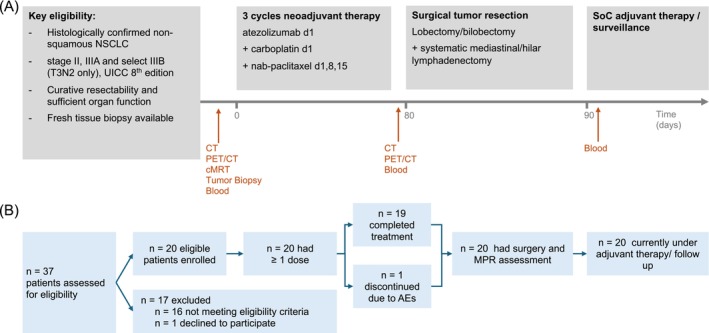
Trial design. (A) Clinical trial design including key eligibility criteria. (B) Patient deposition during the phases of the trial including screening, preoperative immunotherapy and curative resection. AE, adverse event; CT, Computed tomography; MPR, major pathologic response; NSCLC, non‐smallnon–small cell lung cancer; PET, positron emission tomography; SoC, standard of care.

The IReP trial is an open‐label, single‐arm, prospective, monocentric phase II trial (Figure 1A). Patients with previously untreated, pathologically confirmed, non‐squamous NSCLC in stage II, IIIA and select IIIB (T3N2 only) according to the 8th edition of the Union for International Cancer Control (UICC) staging system, considered to be resectable after investigator assessment, were eligible for participation. Patients were required to have an Eastern Cooperative Oncology Group (ECOG) performance‐status score of 0 or 1, age ≥ 18 years, adequate lung and cardiac function for intended lung resection, and the willingness and ability to provide written informed consent and to comply with the study protocol and planned procedures. Full inclusion and exclusion criteria can be found in supplementary methods. Before inclusion in the study, a pre‐treatment tumor biopsy and liquid biopsy were required in all patients for correlative biomarker studies.

Following inclusion, all patients were treated with atezolizumab administrations in combination with chemotherapy (carboplatin/nab‐paclitaxel) for 3 three‐weekly cycles. After completion of the neoadjuvant treatment, a non‐invasive tumor assessment by [18] F‐FDG PET‐CT was performed before curative intent surgery. Patients underwent standard of care surgery with either lobectomy or bilobectomy and postoperative medical therapy and/or radiotherapy if clinically indicated. Surgery and postoperative treatments were not part of the clinical study intervention. All patients will be followed up within standard of care with consecutive sampling of peripheral blood mononuclear cells (PBMC), serum, and plasma for later biomarker studies.

### 2.2 Assessments and Endpoints

The primary endpoint was MPR, assessed in routine histopathologic evaluation of surgical specimen. As defined by Travis et al., a reduction of viable tumor to less as or equal to 10% in the tumor bed was considered MPR [22]. Secondary endpoints included response rates according to RECIST v1.1 criteria [23] and response rates determined by delta PET‐activity. Baseline assessments furthermore included the collection of tumor tissue samples by CT‐guided transthoracic biopsy in all patients, performed as previously described in detail, to investigate the underlying biologies of response [24].

Safety assessments included monitoring and recording adverse events (AEs) and patient‐reported quality of life outcomes. AEs were graded according to the 5‐grade scale defined in the CTCAE V 5.0. Patient‐reported quality of life data was assessed according to the EORTC QLQ‐30 and QLQ‐LC13 questionnaires [25, 26]. Oncogenic driver mutations (e.g., in EGFR, KRAS, BRAF, etc.) were investigated based on sequencing data from routine diagnostics at the Institute of Pathology Heidelberg using small‐scale NGS panels (*n* = 31–54 genes). PD‐L1 staining was performed within clinical routine care at the Institute of Pathology according to current recommendations for lung cancer. The analysis of survival outcomes as well as correlative biomarker studies will be reported once the predefined follow‐up period of 24 months is completed for all trial participants according to the study protocol.

### 2.3 Sample Size Calculation

The sample size calculation was based on MPR as primary endpoint, the assumption of an MPR rate of 22% for chemotherapy alone [21] and accordingly a null hypothesis as H0: MPR ≤ 0.22. An improvement in the MPR rate beyond 22% by the carboplatin/nab‐paclitaxel/atezolizumab regimen was postulated. An MPR rate of 50% was considered a clinically relevant improvement. A sample size of 19 evaluable participants was required to allow to reject the null hypothesis using a one‐sided exact binomial test at level α = 5% with a power of 80% if the true MPR rate was 50%. The sample size calculation was done according to A ´Hern 2001 [27]. Assuming a drop‐out rate of 5%, *n* = 20 participants needed to be enrolled.

### 2.4 Statistical Analyses

A clinical data management system was used for data collection using an electronic CRF (eCRF), ensuring all entries in the eCRF were verifiable by source documents. All data was summarized using descriptive statistics for demographic characteristics and perioperative outcomes, reporting categorical data as counts and percentages and continuous data as median and interquartile range (IQR). A one‐sided exact binomial test was used to test whether the MPR rate achieved in this trial was higher than an MPR rate of 22%, considered insufficient. The Wilcoxon signed rank test was used to assess changes in outcome measures pre‐ and post‐treatment. The relationship between radiologic response and pathologic response (the proportion of residual viable tumor) was assessed using linear regression. Statistical analyses were performed using the R (version 4.5.2, R Foundation for Statistical Computing) and Prism (version 10, Graphpad Software Inc.).

## 3 Results

### 3.1 Patients and Treatment

From April 2021 to June 2024, 20 eligible patients were enrolled in the trial (Figure 1A,B). Baseline demographic and pathologic characteristics of the study cohort are given in Table 1. Patients included 12 (60.0%) female and 8 (40.0%) male patients, with a median age of 60.5 years (IQR 58.5–65.8) and a history of smoking in 16 patients (80.0%). All patients had histologically confirmed pulmonary adenocarcinoma. Clinical stage at screening according to the 8th edition of the UICC TNM staging system was stage IIA in one patient (5.0%), stage IIB in 7 patients (35.0%) and stage IIIA in 12 patients (60.0%). 20 patients (100%) received at least one dose of the neoadjuvant chemoimmunotherapy regimen of Atezolizumab, Carboplatin and nab‐Paclitaxel, and 19 patients (95.0%) received the full course of 3 cycles. The reason that one patient did not receive cycle 2 and 3 was for serious adverse events including diarrhea and neutropenia after the first cycle. Following the neoadjuvant treatment, all patients underwent non‐invasive assessment of tumor response by CT and PET/CT, followed by anatomical lung resection. The surgical approach was open surgery in 11 patients (55.0%) and minimally invasive surgery (MIS) in 9 patients (45.0%). There were no conversions from MIS to open surgery. Pathologic complete resection (R0) was achieved in all patients.

**TABLE 1:** Baseline characteristics and treatment efficacy.

Variable	Patients (*n* = 20)
Sex, *n* (%)	
Female	12 (60.0)
Male	8 (40.0)
Age, median (IQR)	60.5 (58.5–65.8)
ECOG‐PS, *n* (%)	
0	16 (80.0)
1	4 (20.0)
Smoking status, *n* (%)	
Current	7 (35.0)
Former	9 (45.0)
Never	4 (20.0)
Neoadjuvant Atezolizumab, Carboplatin, nab‐Paclitaxel, *n* (%)	
At least one cycle received	20 (100.0)
At least two cycles received	19 (95.0)
Three cycles received	19 (95.0)
Surgical approach, *n* (%)	
Thoracotomy	11 (55.0)
Minimally invasive	9 (45.0)
c‐TNM Stage (screening), *n* (%)	
IIA	1 (5.0)
IIB	7 (35.0)
IIIA	12 (60.0)
c‐TNM Stage (pre‐surgery), *n* (%)	
IA	7 (35.0)
IB	1 (5.0)
IIA	1 (5.0)
IIB	9 (45.0)
IIIA	2 (10.0)
Tumor response per RECIST, *n* (%)	
CR	0 (0.0)
PR	10 (10.0)
SD	10 (10.0)
PD	0 (0.0)
Nodal status	
pN0	13 (65.0)
pN1	0 (0.0)
pN2	7 (35.0)
p‐TNM stage, *n* (%)	
0	5 (25.0)
IA	4 (20.0)
IB	3 (15.0)
IIA	0 (0.0)
IIB	1 (5.0)
IIIA	7 (35.0)
Pathologic response, *n* (%)	
CPR	5 (25.0)
MPR	9 (45.0)
Non‐MPR	11 (55.0)

Abbreviations: CPR, complete pathologic response; CR, complete response; ECOG‐PS, Eastern Cooperative Oncology Group Performance Status; IQR, interquartile range; MPR, major pathologic response; PD, progressive disease; PR, partial response; RECIST, Response Evaluation Criteria In Solid Tumors; SD, stable disease.

### 3.2 Efficacy

**FIGURE 2:**
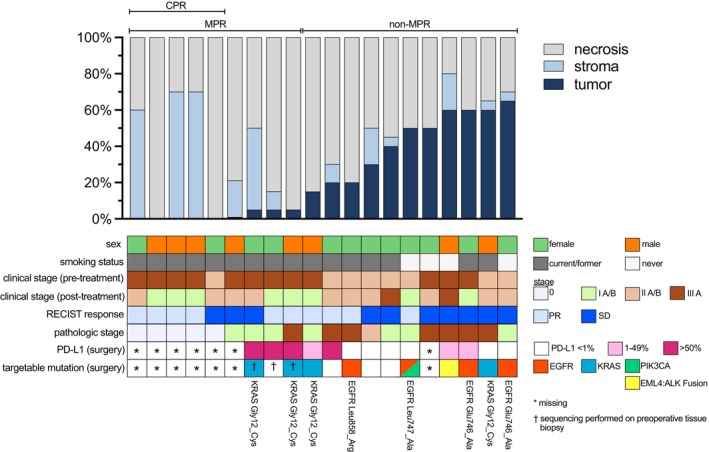
Treatment efficacy. Waterfall plot showing pathological response in surgically resected tumors with important clinical and pathologic features for all patients. PR, partial response; SD, stable disease.

The study met its primary endpoint with a MPR observed in 9 patients (45%, one‐sided 95% confidence interval: 25.9%–100%). A one‐sided exact binomial test indicated that the observed MPR rate was statistically significantly higher than a rate of 22% for chemotherapy alone (*p* = 0.019). Among the 20 patients, 5 patients (25%) exhibited a complete pathologic response (Figure 2).

**FIGURE 3:**
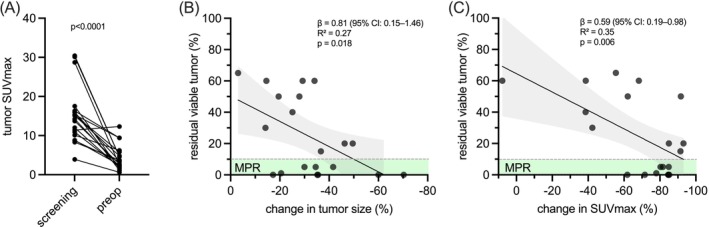
Analysis of the radiologic tumor response. (A) Individual changes in tumor maximal standardized uptake value (SUV_max_) before treatment (screening) and after neoadjuvant chemoimmunotherapy, the *p* value is given for the Wilcoxon signed rank test. (B) Scatter plots with linear regression line and 95% confidence bands show the relationship between the relative change in tumor size on CT. (C) Scatter plots with linear regression line and 95% confidence bands show the relative change in SUV_max_ and residual viable tumor. Each point represents an individual patient. The regression slope (β), coefficient of determination (R [2]) and *p* value summarize the results of a simple linear regression analysis.

Clinical assessment of tumor response according to RECIST criteria demonstrated partial response in 10 (50.0%) patients and stable disease in 10 (50.0%) patients after neoadjuvant therapy. There was no complete remission and no progressive disease (PD). The metabolic tumor response as assessed in [18] F‐FDG PET/CT showed a statistically significant decrease across the overall study cohort with a median SUV_max_ of 14.7 at screening versus 3.4 before surgery (Figure 3A). The predictive value of imaging parameters for pathologic response was investigated by linear regression and correlation analysis. Both the change in tumor size measured in CT and the change in SUV_max_ measured in PET/CT showed a significant but weak relationship with the proportion of residual vital tumor (Figure 3B,C).

### 3.3 Safety

Overall, 278 adverse events were recorded in the neoadjuvant phase, 151 of which were treatment‐related. All of the 20 patients who were enrolled in this trial experienced treatment‐related adverse events (Table 2). 13 patients (65%) had treatment‐related adverse events of grade 3 or higher; 4 of these were considered immune‐related. 6 patients had serious treatment‐related adverse events. Serious treatment‐related adverse events included diarrhea, anemia, leukopenia, autoimmune thyroiditis, polyneuropathy, and atezolizumab infusion‐related orthostatic collapse occurring in one patient each. There were no grade 5 events.

**TABLE 2:** Summary of adverse events (AEs).

All reported AEs	278 events in 20 patients (100.0)
AEs grade 3–4, *n* (%)	23 events in 14 patients (70.0)
AE with fatal outcome (grade 5), *n* (%)	0 events (0.0)
Treatment‐related adverse events (TRAEs)	
All reported TRAEs	151 events in 20 patients (100.0)
TRAEs grade 3–4, *n* (%)	16 events in 13 patients (65.0)
Serious TRAEs, *n* (%)	6 events in 6 patients (30.0)
Diarrhea, *n*	1
Anemia, *n*	1
Leukopenia, *n*	1
Autoimmune thyroiditis, *n*	1
Polyneuropathy, *n*	1
Orthostatic collapse, *n*	1
Immune‐related adverse events (irAEs)	
All reported irAEs	83 events in 18 patients (90.0)
irAEs grade 3–4, *n* (%)	4 events in 4 patients (20.0)
Serious irAEs, *n* (%)	2 events in 2 patients (10.0)
Autoimmune thyroiditis, *n*	1
Orthostatic collapse, *n*	1

*Note:* Serious TRAE/irAE: results in death, is life‐threatening, requires hospitalization or prolongation of existing hospitalization, results in persistent or significant disability or incapacity; AE Grade 1, Mild; asymptomatic or mild symptoms; clinical or diagnostic observations only; Grade 2, Moderate; minimal, local or noninvasive intervention indicated; Grade 3, Severe or medically significant but not immediately life‐threatening, hospitalization or prolongation of hospitalization indicated, disabling; Grade 4, Life‐threatening consequences, urgent intervention indicated. Grade 5; Death related to AE.

Abbreviations: AE, adverse event; irAE, immune‐related adverse event; Pts, patients; TRAE, treatment related adverse event.

### 3.4 Patient‐Reported Outcomes

**FIGURE 4:**
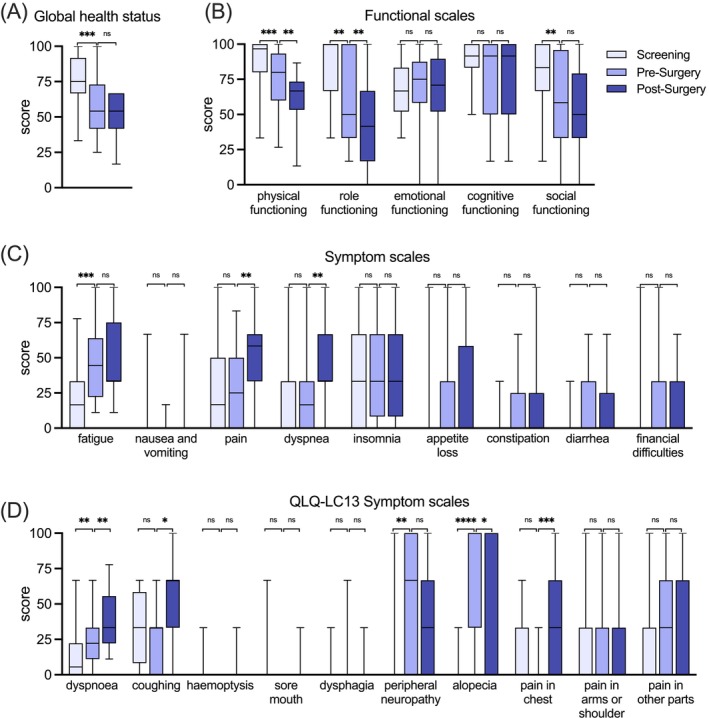
Patient‐reported outcomes. Quality of Life (QoL) outcomes according to the EORTC QLQ‐C30 and QLQ‐LC13 questionnaires. (A) Global health status, a high score represents a high QoL. (B) Functional scales, a high score represents a high/healthy level of functioning. (C) Symptom scales according to QLQ‐C30. (D) Symptom scales according to QLQ‐LC13. A high score represents a high level of symptomatology / problems. Boxplots display the median, the 25% and 75% quartiles and the full data range with asterisks indicating significance between time of Screening, Pre‐Surgery and Post‐Surgery at *p < 0.05, **p < 0.01, ***p < 0.001 and ****p < 0.0001 as determined by the Wilcoxon signed rank test.

Self‐reported quality of life data was assessed using the EORTC QLQ‐30 and QLQ‐LC13 questionnaires at screening, pre‐surgery and after surgery. Global health status significantly decreased during neoadjuvant treatment but did not further decrease during surgery (Figure 4A). Most functional scales documented a continual decline over the whole trial period, including physical functioning, role functioning, and social functioning (Figure 4B). Significant increases in symptomology were observed during the neoadjuvant treatment phase (fatigue, dyspnea, peripheral neuropathy and alopecia), and after surgery (pain, dyspnea, coughing, Figure 4C,D).

## 4 Discussion

To date, limited trial results are available on the efficacy and safety of neoadjuvant chemoIO with atezolizumab in operable lung cancer. The results of the present phase II trial investigating atezolizumab and platinum‐based chemotherapy before curative lung cancer surgery demonstrate encouraging pathologic response rates and a tolerable safety profile. Surgery was feasible in all patients with low morbidity and no mortality. These results further establish neoadjuvant chemoIO with atezolizumab against the background of other PD1/PD‐L1 targeting drugs which have already undergone a more extensive research process. Recent studies have also demonstrated the benefit of other PD‐1 inhibitors like tislelizumab and toripalimab in the neoadjuvant setting, thus adding to the evolving landscape of neoadjuvant IO agents [17, 28, 29].

MPR was defined as the primary endpoint in this trial, because it has been repeatedly identified as a predictive factor for prolonged survival after neoadjuvant chemoIO and is currently employed as the most important surrogate parameter for survival in most clinical trials [30]. Observing an MPR in 45% of patients, we found that neoadjuvant chemoIO with atezolizumab induces a remarkable response similar to other IO agents in combination with chemotherapy, and superior to mono IO [5, 6, 7, 8, 9, 10, 11, 12, 13, 14, 15, 16, 17, 18, 31].

In contrast to pathologic response, evaluating response using radiologic techniques has remained controversial because inflammatory immune‐related reactions can lead to misinterpretation of therapeutic efficacy [32]. Atypical radiologic response patterns may include an initial increase in the primary tumor size followed by a durable response, termed pseudoprogression [33]. Similarly, radiologically abnormal lymph nodes can mimic disease progression, first described in patients treated with neoadjuvant chemoIO in the NEOSTAR trial as the “nodal immune flare” phenomenon, which was associated with an inflamed nodal immune microenvironment [13, 34]. Although iRECIST criteria have been developed to better account for atypical responses and to differentiate between transient pseudoprogression and true progression, the value of radiologic response as surrogate for survival remains ambiguous and less well defined than pathologic response [32, 33] Additionally, PERCIST criteria have been proposed to quantify the metabolic tumor response monitored in PET/CT [35]. In NSCLC, some studies have demonstrated that PET/CT can be a valuable technique for predicting the response to immunotherapy [36, 37]. However in this trial, non‐invasive assessment of tumor response in both CT and PET‐CT had an only moderate correlation to pathologic response, challenging the value of imaging technique for predicting the response to chemoIO [11, 38].

However, non‐invasive assessment of response in imaging in principle has the potential to impact both medical and surgical management by informing treatment decisions like a change of the neoadjuvant regimen or the extent of surgical resection depending on observed response [35, 39]. While our results highlight remaining challenges, future research should aim to improve this by employing combinations of PET/CT with radiomics and deep‐learning based texture analysis, to further identify, understand and characterize response patterns to chemoIO [40, 41]. Especially in the neoadjuvant setting, clinical trial designs have the unique opportunity to correlate imaging parameters with pathologic outcomes, informing response‐based therapeutic strategies.

Treatment response must of course be weighed in the context of safety and toxicity that is potentially augmented by the combination of IO and chemotherapy [42]. In recent phase III trials, TRAEs leading to treatment discontinuation were reported in 10%–13% of patients and TRAEs leading to cancelation of surgery in 1%–6%. Here, discontinuation of chemoIO was necessary in one patient due to diarrhea and neutropenia. However, surgery was feasible in all patients, suggesting an overall well tolerable safety profile. During the neoadjuvant phase, we observed TRAEs in all patients, including 13 (65%) patients with grade 3–4 TRAEs. This is similar to rates reported by Shu et al. for chemoIO but considerably more than for atezolizumab mono, which was associated with only 10% of grade 3–4 TRAEs in the LCMC‐3 trial [10, 20]. Neoadjuvant chemoIO with other PD1/PDL1 targeting drugs report slightly lower rates of grade 3–4 TRAEs ranging from 30%–40%, but larger phase 3 trials are warranted for a robust estimation of TRAE rates associated with the present regimen [5, 6, 7, 8]. Of particular interest in patients who receive neoadjuvant IO are furthermore immune‐related toxicities that may be different from known risks of preoperative chemotherapy. In our trial, 4 patients (20%) experienced irAEs of grade 3–4, which is in line with results from Shu et al. [20] While a potential threat of immune‐related complications in anastomosis healing has been discussed for patients undergoing sleeve lobectomy, recent phase III trials did not show an increased risk with chemoIO and the single patient undergoing arterioplasty in this trial did not experience this complication.

One strength of this trial is the consideration of patient reported outcomes, filling critical gaps that clinical measurements alone cannot capture in the endeavor of a truly patient‐centered care. Similarly to our observations, the Keynote 671 trial has reported a decline in global health status as well as role and physical functioning during the neoadjuvant phase; however, finding no difference between perioperative chemoIO and chemotherapy alone [7]. Encouragingly, both Keynote 671 and CheckMate 77 T reported a return to approximately baseline in quality of life measures after completion of treatment [43, 44]. Our observations are in line with these reports and reflect the adverse impact on global health perception and functional scales from the patient's perspective. Of note, complete resection was safe and feasible for all patients despite the treatment burden of neoadjuvant chemoIO.

During the last decade, minimally‐invasive techniques have become standard of care for the resection of early‐stage NSCLC due to advantages in postoperative pain, functional recovery and complications, while offering similar oncologic efficacy [44]. As experience with MIS grew, both VATS and RATS are now increasingly used in the setting of locally advanced and pretreated NSCLC as well, with about 40% of patients undergoing MIS in the AEGEAN and CheckMate 816 trials [6, 8]. Here, 45% of patients successfully underwent MIS and R0 resection was feasible without intraoperative complications or conversions in all of them. It remains to be determined how these techniques influence long‐term outcomes.

While 45% of patients achieved an MPR, the observed variance in pathologic response highlights a remaining unmet need to better understand and predict the underlying biologic mechanisms of response to chemoIO. Recent studies have explored both mechanisms of immune escape in the tumor microenvironment and explored distinct roles of immune cell subpopulations in chemoIO efficacy, including T‐cells, B‐cells, and innate immunity [45, 46, 47]. Further research should aim to identify mechanisms of action and elucidate the impact of cellular diversity and plasticity of the microenvironment on chemoIO efficacy, ultimately leading to precise prediction, informed treatment strategies, and improved patient outcomes.

This trial has several limitations. First, the single‐arm design without a control group limits definitive conclusions regarding comparative efficacy. Second, the modest sample size, while appropriate for an exploratory phase II trial, restricts precision of effect estimates and precludes robust subgroup analyses in this biologically heterogeneous disease. Third, the study was conducted at a single center, which may affect generalizability. Accordingly, these findings should be interpreted as hypothesis‐generating and warrant confirmation in larger randomized studies.

## 5 Conclusion

Neoadjuvant chemoimmunotherapy with atezolizumab demonstrated encouraging results for patients with resectable non‐squamous NSCLC, achieving a 45% MPR rate at a well tolerable safety profile. Translational biomarker studies, still under analysis, may reveal new avenues for prediction of IO efficacy and non‐invasive assessments of response.

## Author Contributions

**Benedikt Niedermaier:** writing – original draft, data curation, formal analysis, writing – review and editing, investigation. **Tilmann Bochtler:** conceptualization, investigation, methodology, writing – review and editing. **Florian Eichhorn:** investigation, writing – review and editing, formal analysis. **Raffaella Griffo:** investigation, formal analysis, writing – review and editing. **Laura V. Klotz:** investigation, writing – review and editing, formal analysis. **Michael Allgäuer:** investigation, writing – review and editing, data curation. **Albrecht Stenzinger:** investigation, writing – review and editing, formal analysis. **Helge Bischoff:** investigation, writing – review and editing. **Marc A. Schneider:** writing – review and editing, data curation. **Petros Christopoulos:** writing – review and editing, investigation. **Uwe Haberkorn:** investigation, writing – review and editing. **Claus Peter Heussel:** investigation, writing – review and editing. **Inka Zoernig:** investigation, writing – review and editing. **Felix J. F. Herth:** investigation, writing – review and editing. **Michael Thomas:** conceptualization, investigation, writing – review and editing. **Alexander Rölle:** methodology, investigation, writing – review and editing. **Hauke Winter:** investigation, writing – review and editing. **Axel Benner:** methodology, writing – review and editing. **Dirk Jaeger:** conceptualization, funding acquisition, investigation, writing – review and editing. **Martin E. Eichhorn:** conceptualization, investigation, writing – review and editing, supervision, methodology.

## Funding

This trial was funded by the Dietmar Hopp Foundation and supported by the German Center for Lung Research (DZL). The funders had no involvement in the study design; in the collection, analysis, and interpretation of the data; in the writing of the report; and in the decision to submit the paper for publication.

## Ethics Statement

The IREP study was conducted in accordance with the principles of the Declaration of Helsinki. The study protocol was approved by the local ethics committee (IRB Number 838/2020) and registered with the federal authority (Eudra‐CT: 2020–000388‐21). Approvals are available from the corresponding author on reasonable request. Written informed consent for scientific analysis of clinical details, associated medical data, and study‐related results was obtained from all participants before trial inclusion and can be made available on request. An independent data monitoring committee regularly assessed safety data and study conduct.

## Conflicts of Interest

M.E.E. has received advisory/speaker's honoraria from AstraZeneca, Bristol‐Myers Squibb, MSD, Roche, and Intuitive Surgical Inc. T.B. has worked as study oncologist for the CUPISCO trial, which was sponsored by Roche. He has received remuneration for this work for the benefit of his employer, and reimbursement for study‐related travels. He has also received research support from Roche. F.E. received lecture/consulting fees and travel/speaker honoraria from Roche, BMS, AstraZeneca, Daiichi, MSD, and Regeneron Pharmaceuticals. M.A. has received compensation for advisory board at AbbVie and speaker's honoraria from Boehringer Ingelheim. A.S. is member of the advisory board/speaker's bureau at Aignostics, Amgen, Astellas, AstraZeneca, Bayer, Beigene, Bristol Myers Squibb, Eli Lilly and Company, Illumina, Incyte, Janssen, Jazz Pharmaceuticals, Johnson&Johnon, Leo Pharma, Merck Sharp & Dohme, Novartis, Pfizer, Qlucore, QuiP, Sanofi, Servier, Taiho, Takeda, and Thermo Fisher Scientific. He has received grants from Bayer, Bristol Myers Squibb, Chugai, and Incyte. P.C. has received research funding from AstraZeneca, Amgen, Merck, Novartis, PharmaMar, Roche, and Takeda; speaker's honoraria from AstraZeneca, Gilead, Johnson, and Johnson, Merck, Novartis, Pfizer, Roche, Takeda, and ThermoFisher; support for attending meetings from AstraZeneca, Daiichi Sankyo, Eli Lilly, Gilead, Johnson&Johnson, Merck, Novartis, Pfizer, and Takeda; and personal fees for participating in advisory boards from AstraZeneca, Boehringer Ingelheim, Chugai, MSD, Novartis, Johnson & Johnson, Pfizer, Roche, and Takeda; all outside the submitted work; research funding, advisory board, and speaker's honoraria from Roche. C.P.H. owns stock in GSK and has received consulting fees from Schering‐Plough, Pfizer, Basilea, Boehringer Ingelheim, Novartis, Roche, Astellas, Gilead, MSD, Lilly, Intermune, Fresenius, and Voiant; research funding from Siemens, Pfizer, MeVis, Boehringer Ingelheim, and Exscientia; lecture fees from Gilead, Essex, Schering‐Plough, AstraZeneca, Lilly, Roche, MSD, Pfizer, Bracco, MEDA Pharma, Intermune, Chiesi, Siemens, Covidien, Pierre Fabre, Boehringer Ingelheim, Grifols, Novartis, Basilea, Bayer, and Sanofi. M.T. received consultancy fees from Amgen, AstraZeneca, Beigene, Bristol‐Myers Squibb, Boehringer Ingelheim, Daiichi Sankyo, Gilead Sciences, GlaxoSmithKline, Johnson&Johnson, Lilly, Merck, MSD, Novartis, Pfizer, Pharmamar, Pierre Fabre, Regeneron, Roche, Sanofi, Takeda and received research grants from AstraZeneca, Bristol‐Myers Squibb, Johnson&Johnson, Merck, Pharmamar, Roche and Takeda. The remaining authors have no conflicts of interest to declare.

## Supporting information


**Data S1:** ijc70508‐sup‐0001‐Supinfo1.pdf.

## Data Availability

The data underlying this article will be shared on reasonable request to the corresponding author.
