# Supplementary material for: Neoadjuvant Atezolizumab and Chemotherapy for Non‐Squamous Non‐Small Cell Lung Cancer: Efficacy and Safety Results of an Open‐Label, Single‐Arm, Phase II Trial
Source: Int J Cancer. 2026 Apr 19;159(6):1510–20. doi: 10.1002/ijc.70508 (PMC13397192; doi:10.1002/ijc.70508)
Supplement: Supplementary file 1 — Data S1: ijc70508‐sup‐0001‐Supinfo1.pdf. [file IJC-159-1510-s001.pdf]

Neoadjuvant atezolizumab and chemotherapy for non-squamous non-small cell lung cancer: Efficacy and safety results of an open-label, single-arm, phase II trial

Benedikt Niedermaier, Tilmann Bochtler, Florian Eichhorn, Raffaella Griffo, Laura V. Klotz, Michael Allgäuer, Albrecht Stenzinger, Helge Bischoff, Marc A. Schneider, Petros Christopoulos, Uwe Haberkorn, Claus Peter Heussel, Inka Zoernig, Felix J.F. Herth, Michael Thomas, Alexander Rölle, Hauke Winter, Axel Benner, Dirk Jaeger, Martin E. Eichhorn

Supplementary Methods

Inclusion Criteria

- Willing and able to sign a written informed consent form (ICF)
- Informed consent, patients age ≥ 18-year-old including, signed and dated
- Eastern Cooperative Oncology Group (ECOG) performance status 0 or 1
- Histologically confirmed NSCLC of non-squamous histology, cStage II, IIIA or select IIIB (T3N2 only); for T-status ≤ T3 allowed; for N2 patients only IIIa1-3 Robinson classification allowed
- Deemed surgically resectable with curative intent by an attending thoracic surgeon after adequate staging including PET-CT
- Adequate lung and cardiac function for intended lung resection according to German S3 regulation
- Radiologically measurable disease as defined by response evaluation criteria in solid tumors RECIST v1.1
- Sufficient availability of the tissue sample from primary tumor before start of neoadjuvant treatment
- Females of child-bearing potential must agree to use, and be able to comply with, effective contraception (</=1% failure rate annually) without interruption, 28 days prior to starting therapy (including dose interruptions), and while on study medication or for a period of 120 days after the last dose of study medication
- Females must have a negative serum pregnancy test (β -hCG) result at screening and agree to ongoing pregnancy testing during the course of the study, and after the end of study therapy.
- Male subjects must practice true abstinence or agree to use a condom during sexual contact with a pregnant female or a female of childbearing potential while participating in the study, during dose interruptions and for 6 months following treatment discontinuation, even if he has undergone a successful vasectomy.
- Adequate renal, hepatic, and bone marrow function as defined below

- Absolute neutrophil count (ANC) > 1500/µl
- Platelet count ≥ 100000/µl
- Hemoglobin ≥ 9 g/dl (can be post-transfusion)
- International normalized ratio (INR) ≤ 1.4 in patients not receiving anticoagulation; for patients receiving respective anticoagulation an INR ≤3.0 allowed
- Activated partial thromboplastin time (aPTT) ≤ 1.5 times upper limit of normal (ULN) in patients not receiving anticoagulation; for patients receiving respective anticoagulation a PTT ≤2.5 x ULN allowed
- Bilirubin < 1.5 times x ULN (for patients with known Gilbert disease Bilirubin ≤ 3 times x ULN allowed)
- ALT and AST < 2.5 times x ULN
- Creatinine ≤ 1.5 x ULN or calculated creatinine clearance > 60 ml/min for subjects with creatinine levels > 1.5 x ULN; for patients meeting the criterion of creatinine ≤ 1.5 x ULN also a calculated creatinine clearance of > 30 ml/min is mandatory

Exclusion Criteria

- Illness or condition that may interfere with a patient's capacity to understand, follow, and/or comply with study procedures
- Treatment in any other clinical trial within 30 days before screening.
- NSCLC Stage cT4
- NSCLC stage cN3 or cN2 IIIA4 (bulky or fixed multi-station N2 disease) according to Robinson classification
- NSCLC of squamous cell histology
- Any prior therapy for lung cancer (including systemic therapy, radiotherapy or major surgery)
- Malignancies other than NSCLC within 5 years prior to study inclusion with the exception of malignancies with a negligible risk of metastasis or death (5-year OS > 90%) like localized prostate cancer, ductal carcinoma in situ, adequately treated carcinoma in situ of the cervix, Stage I uterine cancer or non-melanoma skin carcinoma
- History of allogeneic tissue / solid organ transplant or allogeneic stem cell transplantation
- Patients with active hepatitis B or C infections or a history of HIV infection
- Pregnant or lactating women
- Active autoimmune disease or history of severe autoimmune disease or immunodeficiency or a syndrome that requires systemic steroids or immunosuppressive agents
- The following exceptions are granted:
    - patients with vitiligo, eczema, lichen simplex or resolved childhood asthma/atopy
    - subjects requiring intermittent use of bronchodilatators or local steroid injections
    - patients with hypothyreoidism stable on hormone replacement
- Treatment with systemic immunosuppressive medications (including but not limited to prednisone, cyclophosphamide, azathioprine, methotrexate, and anti-tumor necrosis factor (anti-TNF) agents) within 2 weeks prior to Cycle 1, Day 1 (except low-dose steroids for adrenal failure or emesis prophylaxis)

- History of idiopathic pulmonary fibrosis, interstitial lung disease, organizing pneumonia, drug-induced pneumonitis, or evidence of active pneumonitis on screening chest Computed Tomography (CT) scan
- Prior treatment with cluster of differentiation 137 (CD137) agonist or immune checkpoint blockade therapies, anti-programmed-death-1 (anti-PD-1), and anti-PD-L1 therapeutic antibody
- Live vaccine within 30 days prior to first dose of trial treatment
- Cerebrovascular accident within the past 6 months
- Severe infection or significant traumatic injury within the past 4 weeks
- Clinically significant history of cardiovascular disease, including any of the following:
    - Myocardial infarction or unstable angina within the past 6 months
    - New York Heart Association class II, III-IV congestive heart failure
    - Poorly controlled cardiac arrhythmia despite medication, except rate-controlled atrial fibrillation
- Known allergy or hypersensitivity to any component of the chemotherapy regimen
- Patients who have been incarcerated or involuntarily institutionalized by court order or by the authorities, as well as patients who are unable to consent because they do not understand the nature, significance and implications of the clinical trial
